# Supplementary material for: An Investigation of the Influence of Age and Saliva Flow on the Oral Retention of Whey Protein and Its Potential Effect on the Perception and Acceptance of Whey Protein Beverages
Source: Nutrients. 2020 Aug 19;12(9):2506. doi: 10.3390/nu12092506 (PMC7551043; doi:10.3390/nu12092506)
Supplement: Supplementary file 1 [file nutrients-12-02506-s001.pdf]

Supplementary Materials: The following are available online at www.mdpi.com/xxx/s1, Table S1. Summary of gender and medication of volunteers in both studies. Figure S1: Influence of visit on protein concentration in saliva samples post whey protein beverage (WPB) consumption by sample and timepoints (WPCU: unheated WPB; WPCH: heated WPB). Figure S2: Additional factors influencing protein concentration in saliva samples post whey protein beverage consumption, with relevant *p* value above each category. Table S2: Summary of baseline protein concentration (mg/ml) in saliva samples in both studies. Table S3: Additional factors influencing volunteers (n = 84) liking, effort to consume, attribute perception and appropriateness of attribute level (Just-About-Right, JAR) mean ratings of whey protein beverages. Table S4: Volunteers counts of whey protein beverage (WPB) preference and consumption habits. Table S5: Summary of volunteers (n = 84) whey protein beverage comments.

Table S1. Summary of gender and medication of volunteers in both studies.

	**Pilot Study** (n = 22)				**Main Study** (n = 84)							
	Gender				Gender				Medication			
	Male		Female		Male		Female		Yes		No	
	n	%	n	%	n	%	n	%	n	%	n	%
Total	5	23	17	77	31	37	53	63	19	23	65	77
Younger Adults	5	23	17	77	12	29	30	71	0	0	42	100
Older Adults	0	0	0	0	19	45	23	55	19	45	23	55

n and % reflect number and percentage in each contributing group. Pilot study: all younger adults without any medication and main study (younger adults: n = 42; older adults: n = 42).

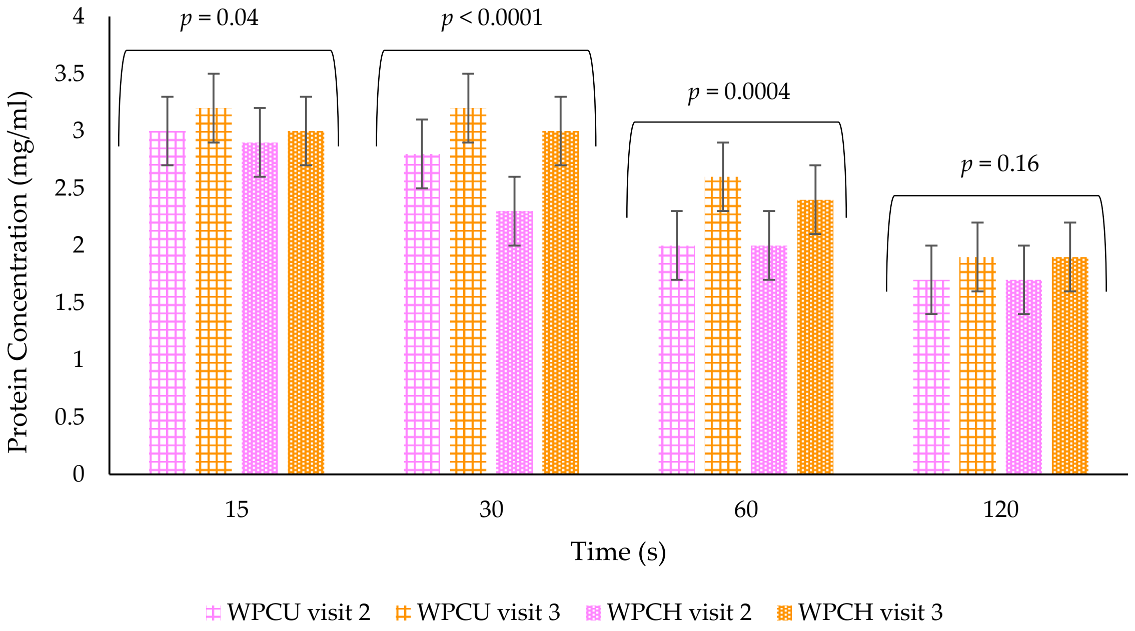

Figure S1. Influence of visit on protein concentration in saliva samples post whey protein beverage (WPB) consumption by sample and timepoints (WPCU: unheated WPB; WPCH: heated WPB). Values are expressed as LSM estimates ± standard error from SAS output. Significant differences ($p < 0.05$) were reported between sample*visit*timepoints with relevant p value above each timepoint and visit 2 (n = 84) and visit 3 (n = 82).

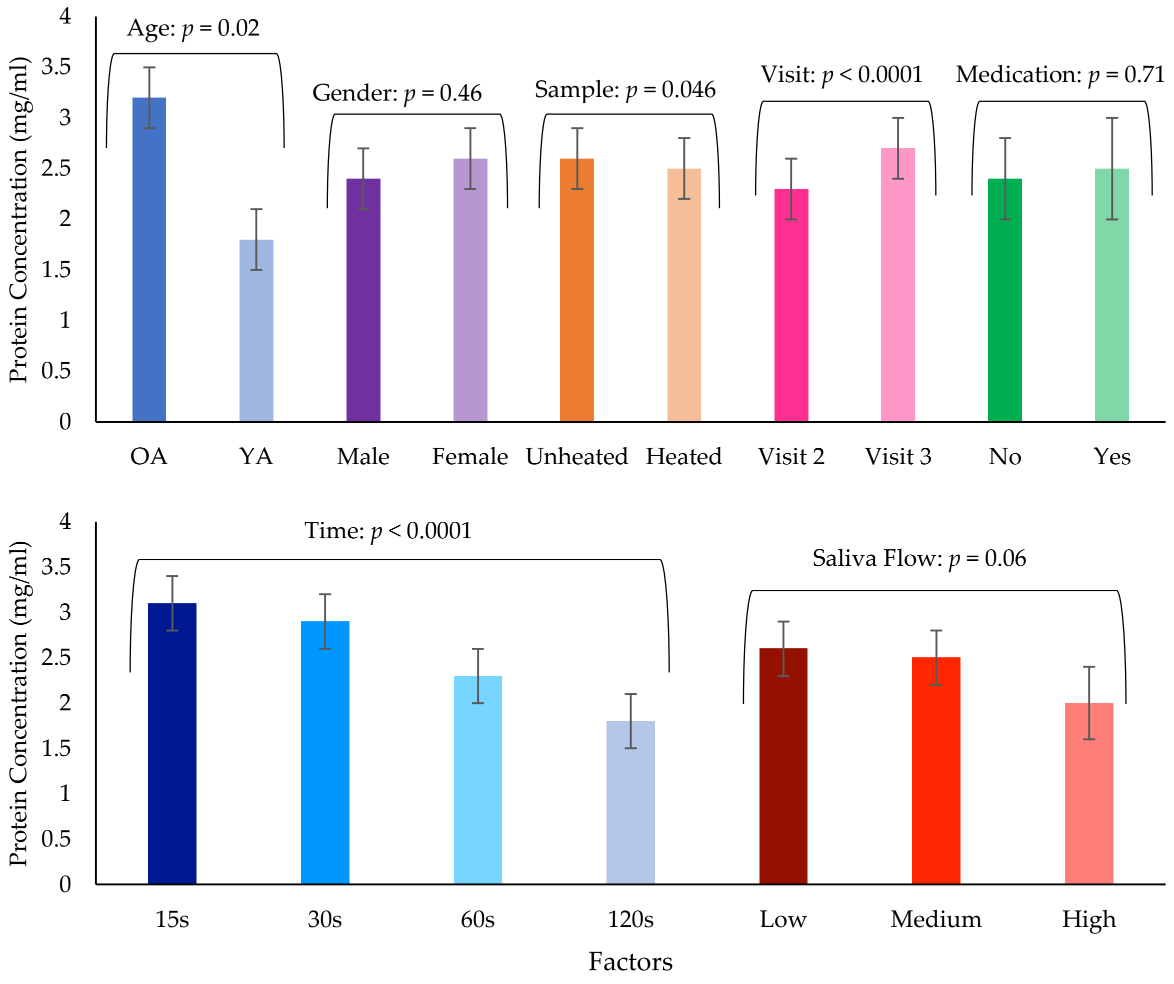

Figure S2. Additional factors influencing protein concentration in saliva samples post whey protein beverage consumption, with relevant *p* value above each category. Values are expressed as LSM estimates ± standard error from SAS output and a higher value would suggest greater adhesion.

Table S2. Summary of baseline protein concentration (mg/ml) in saliva samples in both studies.

	**Pilot Study** (n = 22)			**Main Study** (n = 84)						
	Overall	Gender		Age		Gender		Visit or Rep		
		Male	Female	YA	OA	Male	Female	1	2	3
		(n = 5)	(n = 17)	(n = 42)	(n = 42)	(n = 31)	(n = 53)	(n = 84)	(n = 84)	(n = 82)
USF	0.78 ± 0.1	0.79 ± 0.03	0.78 ± 0.03	1.0 ± 0.1	1.6 ± 0.1*	1.3 ± 0.1	1.2 ± 0.09	1.2 ± 0.09	1.2 ± 0.09	1.3 ± 0.09
SSF	0.78 ± 0.1	0.80 ± 0.03	0.78 ± 0.03	1.0 ± 0.07	1.0 ± 0.07	1.0 ± 0.08	1.1 ± 0.06	1.0 ± 0.06	1.0 ± 0.06	-

Values are expressed as LSM estimates ± standard error from SAS output and significant differences ($p < 0.05$) between groups are denoted by *. USF: unstimulated saliva flow; SSF: stimulated saliva flow; YA: younger adult; OA: older adult. Visit only applied to unstimulated saliva and rep only applied to stimulated saliva.

Table S3. Additional factors influencing volunteers (n = 84) liking, effort to consume, attribute perception and appropriateness of attribute level (Just-About-Right, JAR) mean ratings of whey protein beverages.

	**Medication**		**Gender**	
	No	Yes	Male	Female
	(n = 65)	(n = 19)	(n = 31)	(n = 53)
Overall Liking	4.0 ± 0.2	2.9 ± 0.4*	3.4 ± 0.3	3.5 ± 0.3
Easiness to Drink	4.0 ± 0.1	3.6 ± 0.2	3.9 ± 0.2	3.7 ± 0.1
Easiness to Swallow	4.1 ± 0.1	3.9 ± 0.2	4.2 ± 0.1	3.8 ± 0.1*
Mouthdrying	21.1 ± 3.0	23.8 ± 5.3	21.0 ± 4.2	23.0 ± 3.8
Sweetness	6.2 ± 0.9	7.2 ± 1.7	7.4 ± 1.3	6.1 ± 1.2
Thickness	10.8 ± 1.7	16.3 ± 2.9	15.3 ± 2.3	11.8 ± 2.0
JAR Flavour	2.3 ± 0.1	2.5 ± 0.2	2.5 ± 0.2	2.3 ± 0.1
JAR Thickness	2.4 ± 0.1	2.6 ± 0.2	2.7 ± 0.1	2.3 ± 0.1

Values are expressed as LSM estimates ± standard error from SAS output and significant differences ($p < 0.05$) between groups are denoted by *.

Table S4. Volunteers counts of whey protein beverage (WPB) preference and consumption habits.

	**Preference**			**Consumption Habits**	
	WPCU	WPCH	Significance of sample (*p* value)	Yes	No
Total (n = 84)	41	43	0.46	10	74
Younger Adults (n = 42)	27	15	**0.03**	8	39
Older Adults (n = 42)	14	28	**0.03**	2	35

WPCU (unheated WPB) and WPCH (heated WPB). WPB were most frequently consumed at breakfast (n = 9) and nutritional drinks consumption was also recorded (n = 10/84 YA: n = 8/42 and OA: n = 2/42).

Table S5. Summary of volunteers (n = 84) whey protein beverage comments.

	**Flavour Related Comments**									**Texture Related Comments**								
	Positive			Negative			No Comments Provided			Positive			Negative			No Comments Provided		
	Total	YA	OA	Total	YA	OA	Total	YA	OA	Total	YA	OA	Total	YA	OA	Total	YA	OA
WPCU	2	0	2	53	24	29	29	18	11	15	9	6	33	13	20	36	20	16
WPCH	1	0	1	57	23	34	26	19	7	12	7	5	39	17	22	33	18	15

WPCU (unheated WPB) and WPCH (heated WPB). Main study (YA, younger adults: n = 42; OA, older adults: n = 42). Positive refers to refreshing, OK, JAR, smooth and negative refers to aftertaste, metallic, soapy, mouthdrying, bland, neutral, no flavour, horrible, watery and thickness.
